# Neuropeptide-induced modulation of carcinogenesis in a metastatic breast cancer cell line (MDA-MB-231^LUC+^)

**DOI:** 10.1186/s12935-018-0707-8

**Published:** 2018-12-22

**Authors:** Silvia Gutierrez, M. Danilo Boada

**Affiliations:** Department of Anesthesiology, Pain Mechanisms Lab, Wake Forest School of Medicine, Medical Center Boulevard, Winston-Salem, NC 27157-1009 USA

**Keywords:** Neuropeptides, Metastatic cancer, Chemokinesis

## Abstract

**Background:**

Metastatic cancer to bone is well-known to produce extreme pain. It has been suggested that the magnitude of this perceived pain is associated with disease progression and poor prognosis. These data suggest a potential cross-talk between cancer cells and nociceptors that contribute not only to pain, but also to cancer aggressiveness although the underlying mechanisms are yet to be stablished.

**Methods:**

The in vitro dose dependent effect of neuropeptides (NPs) (substance P [SP], calcitonin gene-related peptide and neurokinin A [NKA]) and/or its combination, on the migration and invasion of MDA-MB-231^LUC+^ were assessed by wound healing and collagen-based cell invasion assays, respectively. The effect of NPs on the expression of its receptors (SP [NK1] and neurokinin A receptors [NK2], CALCRL and RAMP1) and kininogen (high-molecular-weight kininogen) release to the cell culture supernatant of MDA-MB-231^LUC+^, were measured using western-blot analysis and an ELISA assay, respectively. Statistical significance was tested using one-way ANOVA, repeated measures ANOVA, or the paired *t*-test. Post-*hoc* testing was performed with correction for multiple comparisons as appropriate.

**Results:**

Our data show that NPs strongly modify the chemokinetic capabilities of a cellular line commonly used as a model of metastatic cancer to bone (MDA-MB-231^LUC+^) and increased the expression of their receptors (NK1R, NK2R, RAMP1, and CALCRL) on these cells. Finally, we demonstrate that NPs also trigger the acute release of HMWK (Bradykinin precursor) by MDA-MB-231^LUC+^, a molecule with both tumorigenic and pro-nociceptive activity.

**Conclusions:**

Based on these observations we conclude that NPs exposure modulates this breast cancer cellular line aggressiveness by increasing its ability to migrate and invade new tissues. Furthermore, these results also support the pro nociceptive and cancer promoter role of the peripheral nervous system, during the initial stages of the disease.

## Background

Degree of pain experienced by patients in metastatic cancer is associated to disease progression and poor prognosis [1]. In most cases, pain with early bone metastasis cannot be explained by tissue damage or the magnitude of the inflammatory process, indicating a neuropathic nature [2–7]. Particularly important to this process are the orthodromic activation of nociceptive sensory afferents to signal pain (peripheral pain sensors) and the antidromic release of their bioactive contents into surrounding tissues (neurogenic inflammation) [8].

This neuro-inflammatory process includes the release of several neuropeptides (NPs) (substance P [SP]), calcitonin gene-related peptide [CGRP] and neurokinin A [NKA]) well recognized for their pro-tumorigenic functions via paracrine and autocrine loops [9–13]. Furthermore, NPs and their receptors are implicated in the acquisition of oncogenic properties and the facilitation of bone marrow metastasis [14–18]. SP bind preferentially to the NK1 receptor (unlike NKA that can bind to both NK1 and NK2 receptors [19]). Concomitantly, CGRP bind to the G Protein-Coupled Receptor complex formed between calcitonin receptor-like receptor (CALCRL) and the receptor activity modifying protein (RAMP-1) for review see Barwell et al. [20].

On the other hand, pain caused by metastatic cancer to bone is unique in severity. Although clearly associated with organ function and tenso-elastic properties, bone nociceptive innervation appears to be particularly prone to cancer activation. It has been observed that some metastatic bone cancer cells release several molecules that can activate nociceptors by modulating Ca^2+^ conductance and triggering a neurogenic response, especially with the release of bradykinin (BK) [21].

These data indicate a potential amplification loop linking cancer cells and sensory nociceptors. The current study focusses on this putative interaction by studying the direct effects of NPs individually and in combination on the metastatic potential of a well characterized human triple negative breast cancer cell line (MDA-MB-231^LUC+^), their effects on the expression of the receptors upon which they act and their action on to cancer cells release of a pro-nociceptive molecular mediator (bradykinin precursor).

## Methods

### Cell culture, reagents and treatments

MDA-MB-231^LUC+^ cells that stably express firefly luciferase gene (#AKR-231, Cell Biolabs, CA, US) were grown at 37 °C and 5% CO_2_ in DMEM media (#11995-065, Gibco by Life technologies, NY, US), containing MEM Non-Essential Amino Acids (#11140-050, Thermo Fisher Scientific, MA, US), 10% fetal bovine serum (FBS) (#F2442, Sigma, MO, US), penicillin/streptomycin (#15140-122, Gibco by Life technologies, NY, US and amphotericin B (#400-104, Gemini Bio-products, Ca, US). Cells were cultured in cell culture media (control) or with SP (#1156), CGRP (#1161), NKA (#1152) (all from Tocris Bioscience, Bristol, UK) or their combination for 24, 48, or 72 h. Solutions were prepared in serum free media and replaced daily. No peptidase inhibitor was used.

### Migration assay

35 × 10^3^ cells were added to culture-inserts (#81176, Ibidi, Munich, Germany) and cultured for 24 h at 37 °C in 5% CO_2_. Afterwards, the insert was removed, and cells were allowed to migrate in FBS free media containing vehicle (media), 1, 10, 100 and 1000 nM of SP, CGRP or NKA (when the cells were separately exposed to each NP [dose–response experiments]) or 100 nM of SP, 100 nM of CGRP, 50 nM of NKA or their combination (when the cells were simultaneously exposed to the three NPs). Images obtained of the wound (three images per treatment group) were taken immediately after removing the insert (0 h) and after 18 h using an Olympus-CK2 inverted microscope (10× phase contrast objective) and a USB camera (Dino-Lite, AM4023X). Gap closure was then measured in five different areas of the gap and their values averaged, using DinoCapture 2.0.

### Invasion assay

250 × 10^3^ cells were added to collagen-based cell invasion chambers (#ECM551, Millipore, MA, USA) and incubated for 24, 48 or, 72 h (three separate experiments, each in duplicate) at 37 °C in 5% CO_2_. FBS (10%) was used as a chemo attractant. Solutions containing the NPs (100 nM of SP, 100 nM CGRP, 50 nM of NKA or their combination) or vehicle (media) were replaced every 24 h. After the treatment was completed, noninvasive cells from the interior of the insert were removed. Inserts containing invasive cells were stained, washed, imaged (using a 10× objective and a Nikon E600 upright microscope equipped with a CCD digital camera), and lysed with the extraction buffer provided in the collagen-based invasion assay (50% of Reagent Alcohol [90% ethanol, 5% methanol and 5% isopropanol] in 50 mM acetic acid, pH 4.5). Dye mixture was transferred to a 96-well plate and absorbance at 560 nm was measured using a spectrophotometer (Epoch Microplate Spectrophotometer, BioTek Instruments Inc, Vermont, USA).

### Western blot

Cultured cells (control and treated with the NPs) were collected, lysed in homogenization buffer (mammalian cell lysis kit (#MCL-1), Sigma, MO, US) containing a protease inhibitor cocktail (1:1000) and centrifuged. Afterwards, supernatants were collected for immunoblotting (three separate experiments). Protein concentration was determined by a protein assay (#5000006, Bio-Rad Laboratories, CA, US). Twenty microgram of total protein were combined with gel loading buffer, heated to 95 °C for 5 min and separated on 10% Tris–HCl gels (#5671034, Bio-Rad Laboratories, CA, USA). Treated groups were loaded next to its corresponding controls at each time point in each gel. The proteins were transferred to PVDF membranes (#162-0177, Bio-Rad Laboratories, CA, USA), blocked in 5% dry milk in PBS and incubated for 2 h at room temperature or overnight at 4 °C with the primary antibodies Anti-CALCRL (1:500, #PA5-50644, Thermo Fisher Scientific, MA, US); Anti-RAMP1 (1:5000, #156575, Abcam, CA, US), Anti-Neurokinin A Receptor (1:1000, #ab124998, Abcam, CA, US); Anti GAPDH (1: 1000, #MAB374, EMD Millipore, MA, US) or Anti-SP Receptor (1: 500, #ABN1369, EMD Millipore, MA, US), respectively. After washing, the membranes were incubated with horseradish peroxidase-conjugated anti-rabbit or anti-mouse secondary antibodies (1:2000, #Sc-2004 or #Sc-2314, respectively, Santa Cruz Biotechnology, TX, US). Signal was visualized using SuperSignal (West Pico or Femto Chemiluminescence Substrate, (#34080 and #34095, respectively, Thermo Scientific, IL, USA) and quantified using an imager (Amersham Imager 600, USA). The ratio of NK1 R, NK2 R, RAMP1, or CALCRL to GAPDH was calculated for each lane and the values of these ratios were normalized to the control group.

### Kininogen (HMWK) release

100 × 10^3^ cells were grown in 24-well plates and pretreated after 48 h of culture for up to 1 h (15, 30 and/or 60 min) in serum-free media with the same NPs and concentrations as described above (three separate experiments). Cell culture supernatants were then collected and for HMWK assay using a commercially available ELISA kit (ELISA kit, ab 189574, Abcam, CA, US). Briefly, after preparing duplicates of HMWK standards and samples, they were mixed with the antibody cocktail and incubated at room temperature for 1 h, following the manufacturer’s instructions. After washing all the samples were reacted with the substrate for 10 min afterwards, the reaction was terminated, and the absorbance was read at 450 nm using a spectrophotometer (Epoch Microplate Spectrophotometer, BioTek Instruments Inc, Vermont, USA). A standard curve was generated and the HMWK concentration calculated. The limit of sensitivity of the assay was 8.7 pg/ml, and the coefficient of variation was 7.4%. The final values per group (samples collected after 15, 30, 60 min of treatment with the NPs) were then compared to the vehicle control group.

### Statistical analysis

All data were analyzed for normal distribution. Data are presented as mean and standard deviation. Statistical significance was tested using one-way ANOVA, repeated measures ANOVA, or the paired *t*-test. Post-*hoc* testing was performed with correction for multiple comparisons as appropriate. Analyses were performed with Origin Lab 9.0. By convention, a two-tailed test was used and *P* < 0.05 was considered significant for all analyses. Dose–response curves were fitted to a nonlinear regression variable slope equation using GraphPad Prism 6.0 (GraphPad Software, Inc, La Jolla, CA, USA). The mean of each curve was calculated from two independent experiments.

## Results

### Modulation of cancer cell migration by NPs

**Fig. 1:**
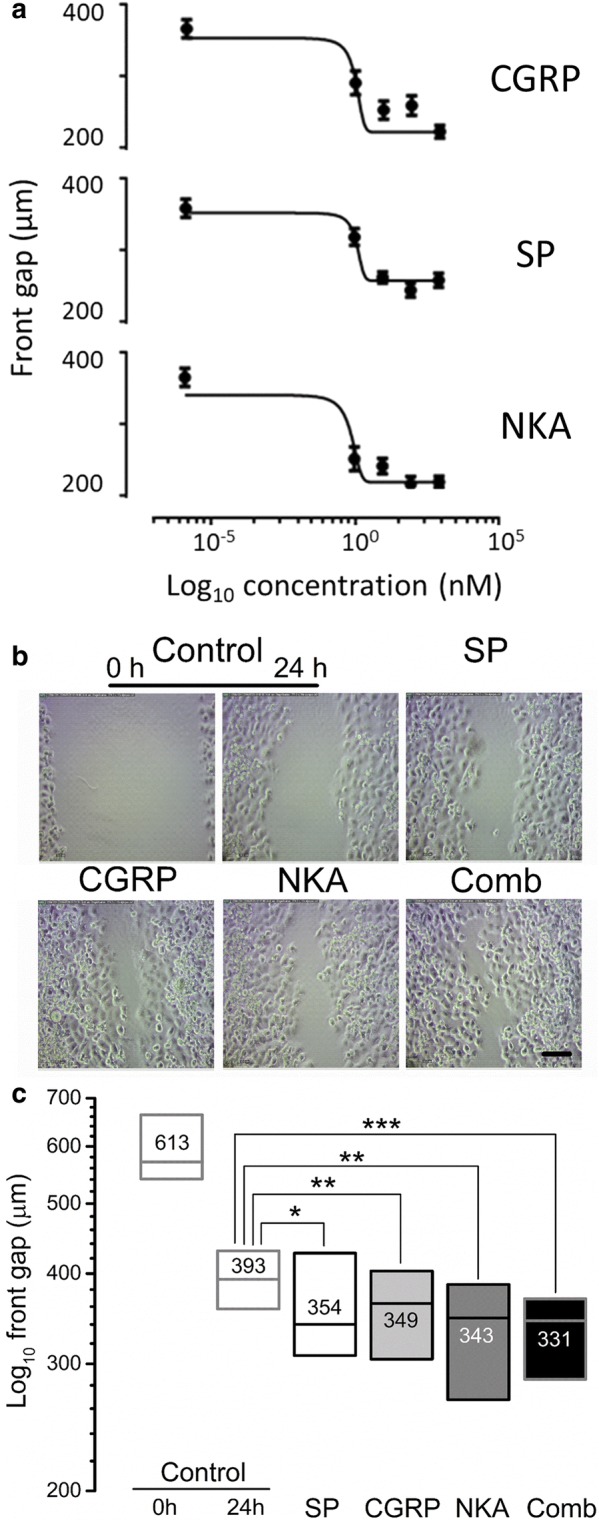
Neuropeptides induced the migration of human metastatic breast cancer cells in the wound-healing assay. **a** NPs induced dose-dependent cell migration in MDA-MB-231^LUC+^ when compared to the control group (R square SP = 0.9752, CGRP = 0.7886 and NKA = 0.9085). **b** Representative images of the wound at 0 and 24 h after substance P (100 nM), CGRP (100 nM), NKA (50 nM) and NP combination, visualized by phase contrast microscopy. Scale bar = 100 µm (bottom left panel). **c** Wound gap decreases in MDA-MB-231^LUC+^ cells 24 h after SP, CGRP, NKA or the combined treatment (Comb) compared to controls. Five independent experiments (each with duplicated samples) and three measurements per image, were performed. Data are presented as median (numbers and horizontal line) with boxes representing the 25 and 75 percentiles, *P < 0.05; **P < 0.01; ***P < 0.001 compared to control treatment

Each NP induced dose-dependent cell migration in MDA-MB-231^LUC+^ compared to the control group (Fig. 1a). The IC_50_ values of SP, CGRP and NKA were 16.46 (from 245 to 348), 10.22 (from 200 to 348) and 4.92 nM (from 198 to 348), respectively. Its curves exhibited an R square coefficient equal to [SP = 0.9752, CGRP = 0.7886 and NKA = 0.9085). Moreover, this effect is also significant (*P* < 0.001) when the cancer cells are simultaneously exposed to the three NPs (Comb: 331 ± 15 µm) (Fig. 1b, c).

### Modulation of cancer cell invasion by NPs

**Fig. 2:**
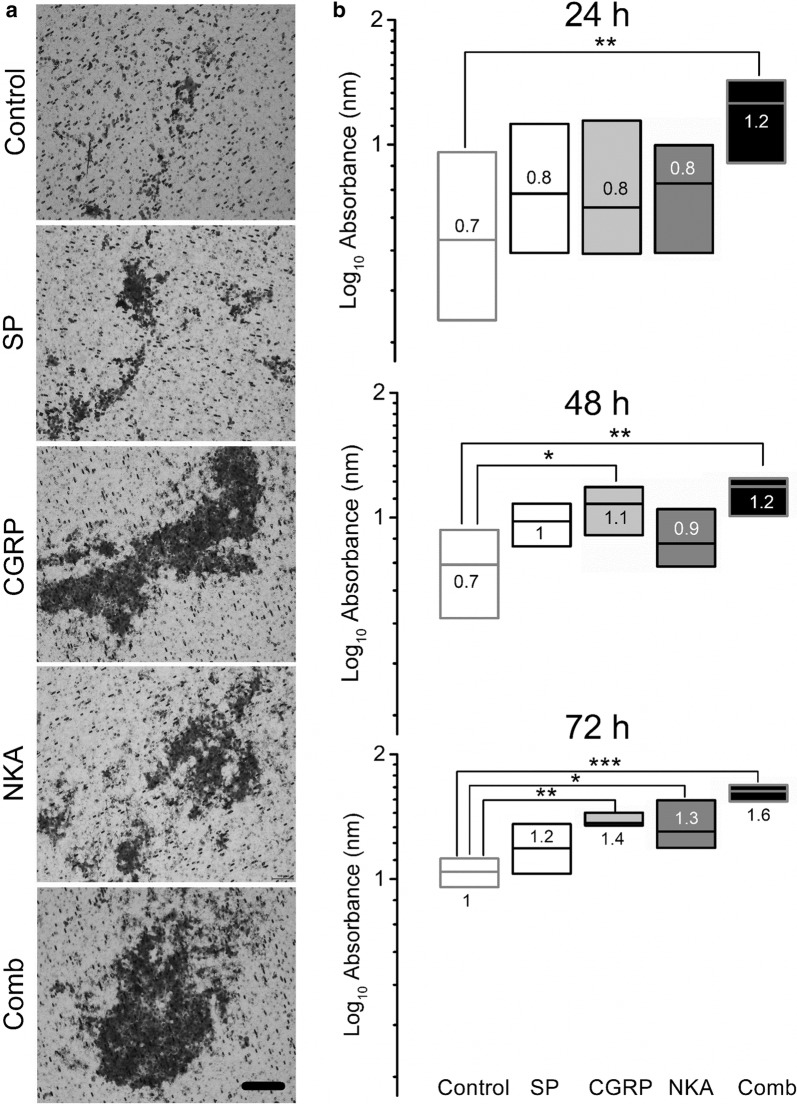
Neuropeptides induced the invasiveness of MDA-MB-231^LUC+^ cells using a a vertical 3D invasion assay. **a** Representative images of the inserts containing the invasive cells at 72 h after substance P (100 nM), CGRP (100 nM), NKA (50 nM) and NP combination (Comb), visualized by transmitted light microscopy. Scale bar = 100 µm (bottom panel). **b** NP time-dependent induced invasion of human metastatic breast cancer cells. NP and its combination increased the invasion of metastatic breast cancer cells after 24, 48 and 72 h (top, center and bottom panel, respectively). Three to five independent experiments (each with duplicated samples) were performed. Data are presented as median (numbers and horizontal line) with boxes representing the 25 and 75 percentiles, *P < 0.05; **P < 0.01; ***P < 0.001 compared to control treatment

NPs alone and in combination also modulated cancer cells invasiveness (Fig. 2). As observed in the migration experiments this modulatory effect differed by NP and was also cumulative. In all three experimental time points (24, 48 and 72 h) the exposure to the combination of all three NPs significantly increased the observed absorbance across the cell lysates (24 h: 1.2 ± 0.15 nm [*P *< 0.01]; 48 h: 1.2 ± 0.11 nm [*P *< 0.01]; 72 h: 1.6 ± 0.05 nm [*P *< 0.001]) compared to control. This is in contrast to the effects of each NP alone, which were restricted to 48 and 72 h after incubation. After 48 h of incubation, only CGRP significantly increased cell lysate absorbance (1.1 ± 0.06 nm [*P *< 0.05]). After 72 h of incubation, CGRP increased absorbance (1.4 ± 0.04 nm [*P *< 0.01]) as did NKA (1.3 ± 0.1 nm [*P *< 0.05]) compared to control. SP, however, failed to differ from control at any experimental time point (Fig. 2a, b).

### Neuropeptide-induced modulation of their cognate receptors on cancer cells

**Fig. 3:**
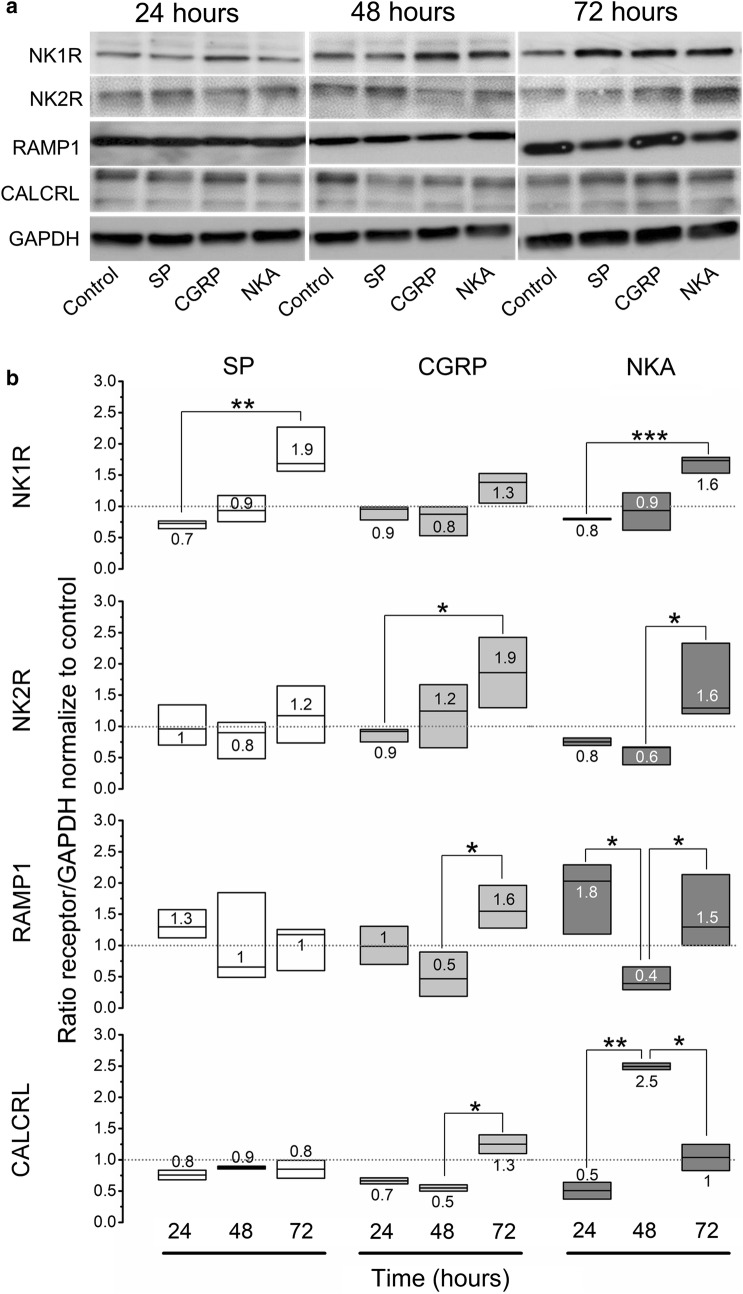
NP time-dependent induced changes in the expression of neurokinin and CGRP receptors in the MDA-MB-231^LUC+^ cells homogenate using Western blot analysis. **a** Representative immunoblots for NK1R (~ 75–100 kDa), NK2R (~ 44 kDa), RAMP1 (~ 17 kDa), CALCRL (~ 45–55 kDa) and GAPDH (~ 37 kDa) from cells homogenates. **b** Changes in the expression of NK-1R, NK-2R, RAMP1 and CALCRL (first, second, third and fourth panel, respectively) in human metastatic breast cancer cells at 24–72 h after NP treatment. Three independent experiments were performed. Data represent NK-1R, NK-2R, RAMP1 and CALCRL/GAPDH ratio, normalized to the mean value for the corresponding control group at 24, 48 or 72 h (punctuated line). Data are presented as median (numbers and horizontal line) with boxes representing the 25 and 75 percentiles, *P < 0.05; **P < 0.01; ***P < 0.001 compared to control or NP treatment group

To further investigate the effects of these NPs on the cancer cell functionality, a quantitative western blot analysis of receptors to each NP was performed. NPs incubation resulted in increased expression of their own receptors and those of other NPs (Fig. 3). As such, CGRP significantly increased expression of both, its receptor and receptor activity modifying protein (RAMP1: 1.6 ± 0.2 [*P* < 0.05] and CALCRL: 1.3 ± 0.2 [*P* < 0.05]) but also NK2R (1.9 ± 0.3 [*P* < 0.05]), after 72 h of incubation. By contrast, SP only increased the expression of NK1R after 72 h of incubation (1.9 ± 0.3 [*P* < 0.01]) without affecting expression of other receptors. NKA increased expression of all four receptors with a significant effect as early as 48 h of incubation. Both NK1R and NK2R expression increased after 72 h of incubation (NK1R: 1.6 ± 0.1 [*P* < 0.001]; NK2R: 1.6 ± 0.4 [*P* < 0.05]). In contrast, NKA altered the expression of CGRP receptors in a more complex fashion, decreasing RAMP1 (0.4 ± 0.1 [*P* < 0.05]) while increasing CALCRL (2.5 ± 0.05 [*P* < 0.01]) after 48 h of incubation. After 72 h these effects were reversed, with increased expression of RAMP1 (1.5 ± 0.3 [*P* < 0.05]) and decreased expression of CALCRL (1 ± 0.2 [*P* < 0.05]) (Fig. 3a, b). No significant change on these receptors’ expression was observed between the control groups at any experimental time point.

### Neuropeptide-induced release of kininogen (HMWK)

**Fig. 4:**
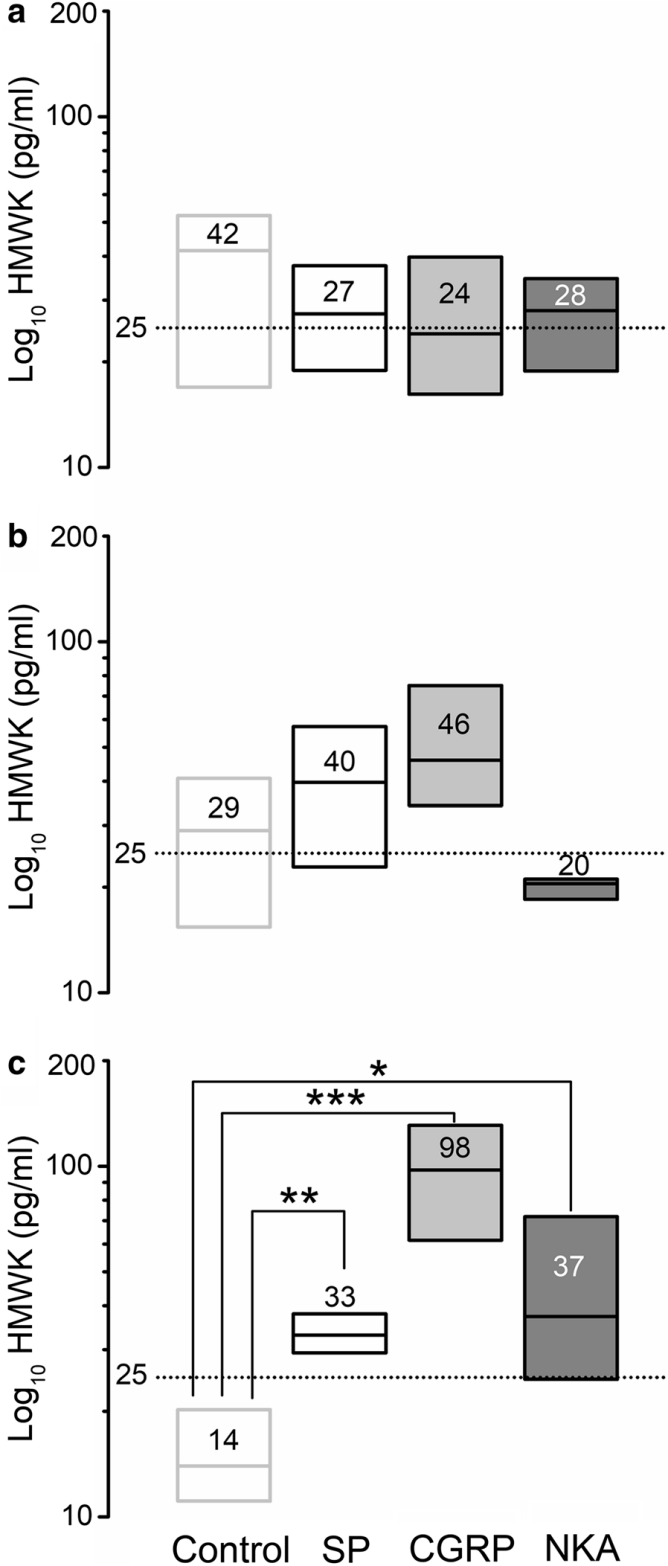
High molecular weight kininogen (HMWK) content changes after NP treatment in human metastatic breast cancer cells supernatant. Cell culture supernatant content was analyzed by enzyme immune-assay. **a**, **b** HMWK content does not changed at 15 or 30 min and increased at 60 min (**c**) after NP treatment, in MDA-MB-231^LUC+^ cells supernatant. Three to five independent experiments were performed. Data are presented as median (numbers and horizontal line) with boxes representing the 25 and 75 percentiles, *P < 0.05; **P < 0.01; ***P < 0.001 compared to control

Finally, we evaluated the effects of NP exposure on the cancer cells acute release of kininogen (HMWK). MDA-MB-231^LUC+^ cells exhibited a basal release of HMWK in absence of exogenous NP (25.4 ± 10 pg/ml) (Fig. 4a, b). HMWK release was increased by exposure to all three NPs after 60 min (SP: 33 ± 4 [*P* < 0.01]; CGRP: 98 ± 20 [*P* < 0.001]; NKA: 37 ± 16 [*P* < 0.05]) (Fig. 4c).

## Discussion

In this study, we report the modulatory effects of NP exposure on chemokinesis (migration and invasion), NP receptor expression and kininogen (HMWK) release of a metastatic human breast cancer cell line (MDA-MB-231^LUC+^). To the best of our knowledge, this is the first report using this extremely aggressive cell line to study the effects of NPs alone and in combination—the latter situation being relevant to the in vivo case with metastasis. Together these observations indicated an NPs-induced increment of this cancer cell line carcinogenic potential. The principal observations and conclusions are: (1) NPs increase MDA-MB-231^LUC+^ chemokinesis, both migration and invasion in this cellular line, (2) NPs alter and primarily increase the expression of all their receptors within the same time frame, and (3) NPs increase the acute release of kininogen (HMWK), molecule with pro-nociceptive and pro-tumorigenic functions, from these cancer cells. Our results further support an interaction between nociceptive sensory neurons and cancer cells that can lead not only to widespread of the disease but also to concurrent extreme pain.

### Neuropeptide modulation of cancer chemokinesis in MDA-MB-231^LUC+^

As indicated above, our results show that NPs are important modulators of MDA-MB-231^LUC+^ migration and invasion. Although novel for this specific human breast cancer line, it’s consistent with other reports about the stimulatory effect of SP in the migration of MDA-MB-468 [22].

The presence of these receptors (NK1R, NK2R, RAMP1, and CALCRL) and the pro-tumorigenic effects of their activating NPs have been demonstrated in a piecemeal fashion across several types of cancer cell lines over the past two decades. For example, NK1R agonists (e.g., hemokinin-1) have been demonstrated to promote migration in non-bioluminescent MDA-MB-231 cells [23, 24] and this is blocked by NK1R antagonists [25]. Moreover, Singh et al. [14] demonstrated that the NK1R is expressed in malignant breast biopsies and the level of its expression has been correlated with the degree of invasion and metastatic potential to the bone of different breast cancer cells types [16]. Similarly, Meshki et al. [26] demonstrated that SP-NK1R signaling induces blebbing on the membrane of HEK293 cells. This mechanism has been identified as the predominant mode used by cancer cell to migrate and infiltrate a variety of tissues (for review see Mierke [27]; Fackler and Grosse [28]).

Less is known regarding pro-tumorigenic effects of the other two NPs (NKA and CGRP) included in our study. Bigioni et al. [29] demonstrated that SP and NKA promote cancer cell proliferation in MDA-MB-231 non-bioluminescent cells. They also showed that both NK1R and NK2R receptor antagonist (MEN 11,467 and MEN 11,420, respectively) inhibited tumor cells proliferation, although chemokinesis was not examined. Relatively little has been reported about the CGRP modulation of tumorigenesis. Some studies suggest a role of CGRP as an angiogenic factor related to the formation of new blood vessels around the growing cancer and a correlation to poor prognosis [30, 31], highlighting the role of neuronal systems in the facilitation of carcinogenesis. Other studies have suggested that CGRP may also have a direct effect on the cancer tumorigenesis. For example, Logan et al. [32] established the importance of RAMP1 receptors in the proliferation and tumorigenicity of human prostate cancer, ultimately leading Austin et al. [33] to argue the significant impact of the tumor-neuron interaction on the disease development and progression.

Although we do not deny a role of NPs in cancer angiogenesis, our results are consistent with the concept that nociceptive activation may have a direct effect on cancer tumorigenesis particularly about cellular chemokinesis [8]. Furthermore, since all three NPs are typically release simultaneously from activated nociceptors, we add to this concept by showing that the combination of these NPs has an overall greater effect on cancer chemokinesis than each alone. Additionally, our results support an important role of neuron-cancer cell interaction at the beginning of the disease and metastasis.

### Neuropeptides receptors modulation in MDA-MB-231^LUC+^

We observed that cancer cells react to NPs by increasing the expression of NP receptors within 72 h, matching the peak time of the chemokinetic effects of NP exposure. There is a large literature about the presence of these receptors (NK1R, NK2R, RAMP1, and CALCRL) in different types of cancer cells. In addition to their role as neurotransmitters, tachykinins and their receptors have been shown to strongly enhance cancer cell growth [34]. Particularly well studied is the tumorigenic role of the SP/NK1R complex in several cancer lines (for review see Muñoz and Coveñas [11, 12, 35, 36]). Moreover, overexpression of the NK1R has been observed in many cancer cell lines [37–39] in both of its isoforms. Interestingly, it has been observed that in addition to the two isoforms NK1R, the amino terminal end of this protein has two glycosylated Asn (N-) sites that affects the functional level of the receptors. As reviewed by Garcia-Recio and Gascon [40], several bands of different molecular weight have been identified, likely due glycosylation. Our observations concur with the description for this receptor (NK1R) and suggest the potential of different affinities for SP yet to be explored in MDA-MB-231^Luc+^.

The role of the NKA/NK2R complex if far less studied. One report noted its presence and potential relevance to the proliferation process of a breast cancer cellular line (e.g., MDA-MB-330) [13] but effects on cellular chemokinesis or interaction with SP/NK1R were not examined. NKA can bind to the NK1R receptor (although with lower affinity than to NK2R) and 50% of its effects in the rat spinal cord can be attributed to NK1R signaling [41]. Signaling of NKA at both to NK1R and NK2R receptors is responsible for its proliferative effect which is reversed by blocking either with NK1R or NK2R [29]. Less is known regarding the chemokinetic effects of CGRP receptors (RAMP1 and CALCRL), although their expression has been demonstrated in several lines of cancer (e.g., prostate and breast cancer) [32]. Rather studies have focused on the angiogenic role of the CGRP/RAMP1/CALCRL complex instead its direct effects on cancer cells. Interestedly, it has been observed that unlike RAMP1, CRLR antibody detected a doublet of bands (~ 55 kDa) that likely correspond with the glycosylated form of the protein (bands also noticed in the current study) [42].

As expected, we demonstrate the presence of these receptors (NK1R, NK2R, RAMP1, and CALCRL) in MDA-MD-231^LUC+^ cancer cells. Like SP, CGRP exposure increases the overall expression of all NPs receptors which may explain the common observation that cancer cells frequently overexpress some of these receptors [37–39]. However, and in the absence of similar studies, its more complicated to explain some of the observed interactions between these NP/Receptors complexes. Kojima and Shimo [43] observed (and later corroborated [44]) a CGRP-mediated enhancement of NK2R expression in the mucosa via myenteric of rodents (guinea pig). According to these authors this interaction is due the CGRP modulation of the release of endogenous tachykinins on these cells leading to formations of an NKA/NK2R complex and its concomitant activation. The same process could explain our observations on the NKA effect on CGRP receptors expression. In the same way, there is evidence that some cancer cells lines (HL60 and BEN) produce endogenous CGRP [45].

### Neuropeptides stimulation of cancer HMW-kininogen release

HMWK is the precursor of bradykinin (BK) via kallikrein–kinin system. Although involved in several physiological processes (e.g., vasodilation, plasma extravasation, bronchoconstriction). BK causes pain by directly stimulating B2 receptors on nociceptors (ultimately inducing the release of SP, NKA, and calcitonin gene-related peptide) as well as sensitizing nociceptors through stimulation of prostanoids production (for review see Dray and Perkinks [46]). Some studies also show that BK may have tumorigenic activity by direct activation of BK receptors (B1R and B2R) [47] which are greatly overexpressed in several types of cancer (particularly in lung, prostate and breast cancer) [48, 49].

Our results show that MDA-MB-231^LUC+^ cells continually release HMWK, which in vivo would result a constant exposure of nociceptors to BK stimulating NP release. The non-bioluminescence MDA-MB-231 indicate the presence of at least one of these receptors (B1R) [47, 50], suggesting they are likely to be present in the bioluminescent line as well. Cancer cell lines vary in their production of HMWK [50–52]. Importantly, in the current study we demonstrate that this basal release of HMWK increase rapidly (within 1 h) after exposure to all three tested NPs.

## Conclusions

NPs enhance chemokinesis (migration and invasion) in MDA-MB-231^LUC+^ cells in vitro and alter, mostly increase expression of their cognate receptors. MDA-MB-231^LUC+^ cells also release HMWK at rest and NPs stimulate increased release of this precursor of BK which would feeds back on nociceptors in vivo, to enhance NP release. Together, these results strongly support the existence of a powerful functional loop of cross-activation between cancer cells and primary sensory nociceptors. Further in vivo studies are required to clarify this concept.

## Abbreviations

BK: bradykinin
CGRP: calcitonin gene-related peptide
HMWK: high molecular weight kininogen
MDA-MB-231^luc+^: breast cancer cell line stably expressing firefly luciferase gene
NKA: neurokinin A
NP: neuropeptide
